# The impact of pre-menarcheal training on menstrual practices and hygiene of Nigerian school girls

**Published:** 2009-06-29

**Authors:** Uzochukwu Uzoma Aniebue, Patricia Nonyelum Aniebue, Theophilus Ogochukwu Nwankwo

**Affiliations:** 1 Department of Obstetrics and Gynecology, University of Nigeria Teaching Hospital, Enugu Nigeria; 2 Department of Community Medicine, University of Nigeria Teaching Hospital, Enugu Nigeria

## Abstract

**Background:**

The menstrual practices of adolescents derive largely from health issues associated with their adjustment to reproductive life. The objective of the study was to assess the effect of pre-menarcheal training on the menstrual and hygiene practices of Nigerian school girls.

**Method:**

A cross-sectional questionnaire-based survey of randomly selected post-menarcheal school girls using a pre-tested, semi-structured questionnaire was done.

**Results:**

The mean age of the school girls was 14.9 ± 1.7 years. Pre-menarcheal training was given to 273 (55.2%) of them. Mothers (74.7%) were the more common source of information. Inappropriate experience of menarche, adverse effect of menstruation on schooling and social life and the use of unhygienic menstrual absorbents were common in girls who had no pre-menarcheal training than those who did.

**Conclusion:**

Lack of timely information results in inappropriate menstrual experiences and poor menstrual hygiene practices. Ways to promote menstrual education and hygiene practices are suggested.

## Background

Menstruation is the cyclical shedding of the inner lining of the uterus, the endometrium, under the control of hormones of the hypothalamopituitary axis. Menarche, or the onset of menstruation, is a landmark feature of female puberty and signals reproductive maturity. Anxiety, fear, confusion, and even depression are frequently reported experiences of menarche [1, 2].

While the anatomy of the genital tract and physiology of menstruation are taught in school in Nigeria, the practical management of menstruation has often been regarded as inappropriate for public discussion [3]. Myths, superstitious beliefs, and cultural taboos substitute appropriate information in the growing child.

Menstrual education is a vital aspect of health education. It is known that attitudes to menstruation and menstrual practices developed at menarche may persist throughout life [4]. The study of the menstrual practices of adolescent girls unveils health issues that affect their adjustment to reproductive life and provides the basis for formulating health education strategies relevant for this crucial period in reproductive life.

The objectives of this study were to ascertain the prevalence of pre-menarcheal training among school girls surveyed in Enugu State, Nigeria and to determine whether hygiene and menstrual practices were influenced by pre-menarcheal training.

## Methods

The survey was administered to consenting post-menarcheal adolescent school girls in Enugu, Nigeria, between March and May 2006. Adolescent girls were defined as those aged 10 and 19 years [5]. Enugu, the capital of Enugu State (southeastern Nigeria), is mostly inhabited by Christians. There are 46 secondary schools in the metropolis comprising of 26 co-educational schools, 12 girls schools and 8 boys schools. At the time of the study, the total student enrolment was 50,822 consisting of 22,089 males and 28,733 females [6]. Permission to carry out the survey was obtained from relevant school authorities. The study was also approved by the Ethical Committee at the University of Nigeria Teaching Hospital, Enugu, Nigeria.

Sample size was determined using a standard formula Z2pq/d2 (Where Z = confidence limit of 95% or 1.96, p = prevalence, q = 1 – p and d = sampling error) [7]. Using a prevalence of 40% for deficiency in knowledge about menstruation from a previous study among Nigerian school girls and a sampling error of 5%, the minimum required sample size was 369 [8]. However, a sample of 500 students was selected to accommodate refusals or non-response.

A local adaptation of the geographical stratification method was used to select the location of the schools studied [9, 10]. Residential areas were stratified into upper, middle and lower classes based on the predominant type of residential building in each area: detached bungalows and storey building for upper class, flats for middle class, and single room houses of multiple-occupation for the lower class. One female school from each stratum was randomly selected and surveyed. The school in the lower class area had 1,931 (all females) students, that in the middle class area had 3,744 (all females) students, and the school in the upper class area had 1,614 (960 girls) students.

The classes in each school were serially numbered and six classes randomly selected using a table of random numbers. Equal numbers of questionnaires were distributed to the selected classes. A brief explanation of the study was done before administering the questionnaires and girls who were pre-menarcheal were informed of their ineligibility for the survey. In each class, consecutive consenting post-menarcheal girls were surveyed beginning from the extreme right hand corner of the first row until the required number was obtained. The survey was carried out during the classes’ free period but within school hours.

The students completed the self-administered, pre-tested, semi-structured English questionnaire in a classroom setting proctored by medical students. The questionnaire had sections on personal data, family background, pre-menarcheal training, age at menarche, attitude to menstruation and menstrual hygiene practices.

Pre-menarcheal training was ranked into “Yes” or “No” and the main outcome measures were the menstrual and hygiene practices of the girls. The other variables studied were (1) Pre-menarcheal training: Respondents were only qualified as having been trained if they responded affirmatively to each of the following questions: (a) Being told about and made to expect the first menstrual bleeding. (b) Having been instructed on how to collect menstrual blood. (c) Having been instructed on how to dispose of the material used to collect menstrual blood. Each question in this segment had a yes or no response. Those who were trained were ranked as “Yes” while those who were deemed untrained as “No”. (2) Menstrual and hygiene practices: Methods used for collection of menstrual blood, frequency of changing the absorbent, method of disposal of the absorbent, and the effect of menses on family, social life, and occupation. (3) Menstrual information: Any oral and written materials related to menstruation and menstrual practices. (4) Menarcheal experience: The feeling of preparedness or otherwise at the time of the first menstrual experience. (5) Menstrual pain: Cyclical lower abdominal pain around the time of menstrual bleeding. (6) Attitude to menstruation: Respondents’ feeling towards the expectation of a menstrual period. It was graded as undesirable (the preference to exist without menses), unprepared (onset of menses usually takes her by surprise) and satisfactory (menses regarded as an acceptable, natural phenomenon that should be expected). (7) Relationship with mother: The extent of closeness to the mother. Relationship with mother was graded as not close, ordinary, close, and very close.

Data entry and statistical analysis were done using SPSS (Statistical Package of Social Sciences) Version 10. Chi square tests were used to assess associations between categorical variables and statistical significance was considered present if the p-value was 0.05 or less. Pearson product moment correlation was used to test for correlation between the educational attainments of the parents and menstrual knowledge of respondents.

## Results

Out of 500 questionnaires, 495 were properly filled yielding a response rate of 99%. The general characteristics of respondents, educational attainment of their parents and the proportion who received pre-menarcheal training are shown in Table 1. The mean age of the students was 14.9 ± 1.7 years and most (98.6%) were Christians. Among the respondents, 52.9% were in senior secondary school and 47.1% were in junior secondary school. Pre-menarcheal training was received by 273 (55.2%) students and the rest (44.8%) had no preparation prior to menarche. Mothers’ and fathers’ highest level of educational attainment were significantly correlated (r = 0.7, P < 0.001). Their mean age at menarche was 12.7+ 1.3 years.

**Table 1:** Characteristics of the 495 secondary school girls surveyed

**Characteristics**	**Frequency**	**%**
**Grade**		
Junior secondary school	262	52.9
Senior secondary school	233	47.1
**Religion**		
Christianity	488	98.6
Islam/ others	7	1.4
**Position in family**		
First Daughter	156	31.5
Middle Daughter	182	36.8
Last Daughter	110	22.2
Only Daughter	47	9.5
**Pre-menarcheal training**		
Yes	273	55.2
No	222	44.8
**Father’s education**		
No formal	28	5.7
Primary	96	19.4
Secondary	133	26.9
Tertiary	238	48.1
**Mother’s education**		
No formal	34	6.9
Primary	74	14.9
Secondary	152	30.7
Tertiary	235	47.5

Mean age of the students was 14.9 ± 1.7 years

The social and other factors influencing the availability of pre-menarcheal training are shown in Table 2. The level of educational attainment of both parents and the sources of information about menstruation were significantly associated with pre-menarcheal training. Girls whose parents received tertiary education and those whose mothers were their main source of information were most likely to have had pre-menarcheal training.

**Table 2:** Factors influencing pre-menarcheal menstrual training

Determinant	Number Trained	%	Unadjusted Odds Ratio (95% CI)^**^	P - value
**Position in family**				
First Daughter (n =156)	88	56.4	Reference	
Middle Daughter (n = 182)	91	50.0	0.63 (0.45 – 0.99)	0.3
Last Daughter (n =110)	64	58.2	1.01 (0.72 – 1.62)	
Only Daughter (n = 47)	30	63.8	1.35 (0.72 – 3.19)	
**Father’s education**				
No formal (n = 28)	12	42.9	Reference	
Primary (n = 96)	36	37.5	0.56 ( 0.42 -0.98)	0.3
Secondary (n = 133)	73	54.9	1.40 (0.98 – 2.10)	
Tertiary (n = 238)	152	63.9	2.80 (1.96 – 4.06)	
**Mother’s education**				
No formal ( n = 34)	13	38.2	Reference	
Primary ( n =74)	38	51.4	1.20 (0.75 – 2.10)	0.3
Secondary (n = 152)	78	51.3	1.20 (0.75 – 2.10)	
Tertiary (n = 235)	144	61.3	2.40 (1.65 - 3.45)	
**Relationship with mother**				
Very close (n = 381)	214	56.2	Reference	0.4
Close (n = 81)	44	54.3	0.80 ( 0.48 –1.28)	
Ordinary (n = 18)	10	55.6	0.80 (0.32 – 2.32)	
Not Close (n = 15)	5	33.3	0.32 (0.08 – 1.04)	
**Age at menarche (years)**				
11 or less (n = 71)	39	54.9	Reference	0.9
12 or more (n = 424)	234	55.2	1.50 ( 0.60 – 1.70)	
**Source of information**				
Mother (n = 354)	204	57.6	Reference	0.02
Other relatives (n = 80)	36	45.0	0.36 ( 0.24 – 0.60)	
Teachers / Health workers (n = 51)	24	47.1	0.42 (0.24 – 0.70)	
Print / Electronic media ( n = 10))	9	90.0	4.50 (0.60 – 96.00)^**^	

** Unadjusted, Unreliable confidence interval.

Inappropriate menarcheal experience, adverse effect of menstruation on schooling and social life, use of unhygienic material as menstrual absorbent and unacceptable methods of disposal for menstrual absorbents were more common in girls who did not have pre-menarcheal training than those who did (Table 3). More girls who had no training disposed of their menstrual absorbents in farms and road side or by recycled them by washing than those who were trained. Girls who did not have pre-menarcheal training also reported more number of severe cases of menstrual pains than those trained. The subsequent menses following menarche were better anticipated and more often reported as satisfactory; changing menstrual absorbent at least thrice daily was more common among those who were trained than those who were not but the differences were not significant.

**Table 3:** Factors influencing pre-menarcheal menstrual training on attitude to menstruation and menstrual and hygiene practices

Attribute	Trained		Untrained		
**Experience at menarche**					
Confusing (n = 106)	44	16.1	62	27.9	
Frightening (n = 143)	69	25.3	74	33.3	é 0.001
Expectant (n = 246)	160	58.6	86	38.7	
**Subsequent menses**					
Undesirable (n = 61)	31	11.4	30	13.5	
Unprepared (n = 83)	49	17.9	34	15.3	0.6
Satisfactory (n = 351)	193	70.6	158	71.2	
**Pain during menses**					
None (n=126)	80	29.3	46	20.7	
Moderate (n =237)	129	47.3	108	48.7	0.05
Severe (n =132)	64	23.4	68	30.6	
**Schooling/social life**					
No Effect (n =135)	91	33.3	44	19.8	
No Sports (n =186)	89	32.6	97	43.7	
Feel Ill (n = 142)	69	25.3	73	32.9	é 0.001
No Domestic Duties (n =22)	18	6.6	4	1.	
No Schooling (n =10)	6	2.2	4	1.8	
**Menstrual absorbent**					
Cloth (n = 45)	21	7.7	24	10.8	
Toilet roll (n =108)	47	17.2	61	27.5	0.006
Sanitary Pad (n = 342)	205	75.1	137	61.7	
**Frequency of change of absorbent/day**					
Once (n = 42)	23	8.4	18	8.1	
Twice (n = 178)	96	35.2	82	36.9	0.9
Three or more (n =275)	154	56.4	121	54.5	
**Absorbent disposal**					
Roadside / Farm (n = 18)	5	1.8	13	5.9	
Washing ( n = 6)	2	0.7	4	1.8	
Toilet (n = 201)	93	34.1	108	48.7	< 0.001^*^
Burning (n = 153)	97	35.5	56	25.2	
Dustbin (n = 117)	76	27.8	41	18.5	

* Chi square result is with Yates correction

## Discussion

In many societies, including Nigeria, menstruation is regarded as very private and is seldom discussed in public or taught openly [1, 2]. More than a third (44.8%) of the respondents in this study had no form of preparation for menarche. This is comparable to 40% previously reported in Ile- Ife Western Nigeria [8]. In Saudi Arabia, ignorance about proper menstrual practices was found in two-fifths of the adolescent surveyed in gynaecological and medical clinics in Riyadh, the capital city [11]. The rate of deficient menstrual training in this study was much lower than the 84% rate observed in a cross sectional study of 250 women aged 15 – 49 years in New Delhi, India, but higher than 25% reported among 250 high school students in Tehran, Iran [12, 13].

Mothers (74.7%) followed by other relatives (13.2%) were the main source of menstrual information in this study. In a related study in Egypt, 92.2% of the girls accessed menstrual information primarily from the mass media [2]. In our study, pre-menarcheal training was significantly related to the educational attainment of the respondent’s parents. This corroborates the finding in a study in western Nigerian which showed that parental education was positively associated with girls’ menstrual knowledge [8]. The educational attainment of parents is expected to influence the economic strength of the family and individuals’ social exposure hence reducing the negative impact of harmful local practices.

The respondents’ relationship with their mothers was not significantly related to pre-menarcheal training. It may hence be speculated that inadequate training of mothers, negligence or reluctance to communicate appropriate menstrual information determines the prevalence of inadequate pre-menarcheal training. Some studies highlight the effectiveness of formal education on female students in relation to health issues [14, 15, 16]. Unfortunately, only 8.8% of the girls in this study received menstrual information from health workers and school teachers. Organized health education complements family life education received at home and serves to correct inappropriate information while stimulating peer dissemination of health information [13].

The consequences of inadequate pre-menarcheal training are multiple. Menarche was described by almost a third (29.9%) of the respondents as frightful. Another 106 (21.4%) girls described their experience as confusing. It has been postulated that the experience at menarche provides the framework for the girl’s latter attitude to menstruation, her body image, and general health behavior [3]. Sixty five percent of the respondents who had satisfactory menarcheal experiences had received pre-menarcheal training. Menstrual pain, which is a common reason for gynaecological consultation [17], is experienced by many women in the early years of their menstrual life [13]. The occurrence of severe menstrual pain and inappropriate menstrual hygiene practices were more common in respondents without pre-menarcheal training than those who had. Training probably modifies perception rather than obliterates menstrual pain. Poor menstrual hygiene practices are known risk factors for genital infections in women and were assessed in this study in terms of sanitary protection method, frequency of changing the menstrual absorbent, and the means of disposal of the absorbent [15]. Most girls (69%) used sanitary pads. The use of cloth and tissue paper pads, however, was more common among girls who had no pre-menarcheal training. Soft sterile pad absorbent changed every four hour regardless of the amount of staining, in the absence of menorrhagia, ensures comfort and prevents the development of offensive odour [2]. Over half of the respondents changed their menstrual absorbent 3 or more times a day and there was no difference by pre-menarcheal training.

An important limitation of this study was recall bias. In addition, the use of geographical stratification of cities is not fully validated because of the broad variation in the construction of cities and factors that determine domicile. The outcome of social classification based on geographical stratification cannot be generalized for all cities and its use in this study was limited to the selection of the school and not for any form of social classification of individual respondents.

Due to wide social and cultural diversity it must be admitted that generalization of the outcome of this study may be limited. It is hoped that future studies would examine the impact of ethnicity, religion and socio- economic diversity on pre-menarcheal training and menstrual practices. Mothers need to be surveyed in order to identify obstacles faced when preparing their daughters for menarche.

## Conclusion

The absence of pre-menarcheal training resulted in inappropriate menstrual experiences and poorer menstrual hygiene practices. The contributions of the health sector, mass media and the formal educational sector in pre-menarcheal training were poor. The media has the important role of promoting the use of sanitary pads and instructions on menstrual health could be incorporated into health education sessions in family planning and antenatal clinics. Women’s associations and professional organizations related to women’s health care should promote public discussion of menstrual health issues through seminars, workshops and conferences. These non-governmental organizations may similarly help in the provision and distribution of sanitary pads at subsidized rates.

Although not directly studied in this survey, it is expected that inclusion of pre-menarcheal training in early secondary school curriculum would enable teachers to address the practical aspects of the management of menses in formal classes. Menstrual health instructions should contain practical discussions on how girls should look after themselves during menses in order to maintain appropriate menstrual and hygiene practices. The issuance of these instructions is best started before menarche. Finally, proper menstrual waste disposal facilities should be provided in schools. It is recommended that toilets be equipped with waste disposal containers and incinerators for sanitary pads. The absence of these conveniences may be at the root of school absenteeism associated with menstruation.

## Competing interests

Authors declared they have no conflicts of interest.

## Authors’ contributions

All authors contributed in the designed, analysis and preparation of the manuscript.

## Acknowledgments

We hereby acknowledge Awkadigwe F.I, Okoro P.I , Onyia U for their assistance in distributing the questionnaires used for this study.
